# Magnetic field dependent small-angle neutron scattering on a Co nanorod array: evidence for intraparticle spin misalignment

**DOI:** 10.1107/S1600576714008413

**Published:** 2014-05-29

**Authors:** A. Günther, J.-P. Bick, P. Szary, D. Honecker, C. D. Dewhurst, U. Keiderling, A. V. Feoktystov, A. Tschöpe, R. Birringer, A. Michels

**Affiliations:** a Physics and Materials Science Research Unit, University of Luxembourg, 162A Avenue de la Faïencerie, L-1511 Luxembourg, Luxembourg; b Institut Laue–Langevin, 6 Rue Jules Horowitz, BP 156, F-38042 Grenoble Cedex 9, France; c Helmholtz-Zentrum Berlin für Materialien und Energie GmbH, Hahn-Meitner-Platz 1, D-14109 Berlin, Germany; d Jülich Centre for Neutron Science JCNS, Forschungszentrum Jülich GmbH, Outstation at MLZ, Lichtenbergstrasse 1, D-85747 Garching, Germany; e Experimentalphysik, Universität des Saarlandes, Postfach 151150, D-66041 Saarbrücken, Germany

**Keywords:** small-angle neutron scattering, magnetism, magnetic materials, nanorods

## Abstract

The structural and magnetic properties of a cobalt nanorod array have been studied by means of magnetic field dependent small-angle neutron scattering (SANS). Measurement of the unpolarized SANS cross section dΣ/dΩ of the saturated sample in the two scattering geometries where the applied magnetic field **H** is either perpendicular or parallel to the wavevector **k**_i_ of the incoming neutron beam allows one to separate nuclear from magnetic SANS, without employing the usual sector-averaging procedure. The analysis of the SANS data in the saturated state provides structural parameters (rod radius and centre-to-centre distance) that are in good agreement with results from electron microscopy. Between saturation and the coercive field, a strong field dependence of dΣ/dΩ is observed (in both geometries), which cannot be explained using the conventional expression of the magnetic SANS cross section of magnetic nanoparticles in a homogeneous nonmagnetic matrix. The origin of the strong field dependence of dΣ/dΩ is believed to be related to intradomain spin misalignment, due to magnetocrystalline and magnetoelastic anisotropies and magnetostatic stray fields.

## Abstract 2

The results of magnetic field dependent small-angle neutron scattering measurements on a cobalt nanorod array are reported. The data provide evidence for the existence of intraparticle spin disorder.

## 1. Introduction   

As a consequence of their interesting magnetic properties, magnetic transition-metal nanorod arrays are attracting much scientific attention (Fert & Piraux, 1999 ▶; Sellmyer *et al.*, 2001 ▶; Kou *et al.*, 2011 ▶; Greaves *et al.*, 2012 ▶). Essentially, it is their pronounced magnetic shape anisotropy which largely determines the magnetization process in these systems and which renders them potential candidates for perpendicular magnetic storage media (Ross *et al.*, 1999 ▶; Greaves *et al.*, 2012 ▶). Owing to the technological relevance of such functional magnetic materials, a better understanding of the microstructure–property relationship is crucial (Goolaup *et al.*, 2005 ▶; Zighem *et al.*, 2011 ▶; Chumakov *et al.*, 2011 ▶).

Small-angle neutron scattering (SANS) is a powerful volume-sensitive technique for probing structural and magnetic properties of such nanorod arrays. In particular, SANS provides access to nanoscale spatial variations of the local orientation and magnitude of the magnetization vector field 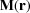 (Wagner & Kohlbrecher, 2005 ▶; Wiedenmann, 2005 ▶; Michels & Weissmüller, 2008 ▶).

Previous SANS studies on ordered arrays of Co and Ni nanowires embedded in 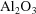 matrices have employed polarized incident neutrons for studying the structural and magnetic correlations (Napolskii *et al.*, 2007 ▶, 2009 ▶; Grigoryeva *et al.*, 2007 ▶; Chumakov *et al.*, 2011 ▶; Maurer *et al.*, 2013 ▶). It is worth mentioning that for Ni nanowires (of average length 50 µm) the validity of the Born approximation has been questioned (Napolskii *et al.*, 2009 ▶), while for Co nanowires an anomalously low magnetic scattering contribution (relative to the nuclear SANS) has been reported (Chumakov *et al.*, 2011 ▶). The non-negligible but relevant influence of magnetostatic stray fields on the magnetization distribution inside the wires has been pointed out by Napolskii *et al.* (2009 ▶) and Maurer *et al.* (2013 ▶).

In this paper, we provide a SANS study of a (short-range-ordered) Co nanorod array using unpolarized neutrons. The focus of our study is on the field dependence of the cross section in the two scattering geometries that have the applied magnetic field either perpendicular or parallel to the wavevector of the incoming neutrons. In particular, the discussion addresses the validity of the standard expression for the magnetic SANS cross section, which assumes uniformly magnetized particles.

## 2. Experimental   

### 2.1. Sample preparation and characterization   

The Co nanorod array was prepared by pulsed electrodeposition of Co into a nanoporous aluminium oxide layer. A detailed description of the synthesis of porous alumina templates and their filling with metals can be found elsewhere (Günther *et al.*, 2008 ▶, 2011 ▶; Klein *et al.*, 2009 ▶); here, we present only a brief outline of the sample preparation. The porous alumina template was synthesized by a two-step anodization process (Masuda & Fukuda, 1995 ▶; Masuda & Satoh, 1996 ▶). The anodization was carried out in 2 *M* sulfuric acid at constant cell voltages of 15 and 20 V (first and second anodization step, respectively). A total charge density of 2 C cm^−2^ during the second anodization and a final treatment of the alumina templates in 0.1 *M* phosphoric acid resulted in an oxide layer thickness of ∼1200 nm, a pore diameter of *d* ≃ 27 nm and a centre-to-centre distance of the pores of 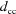 ≃ 48 nm.

**Figure 1:**
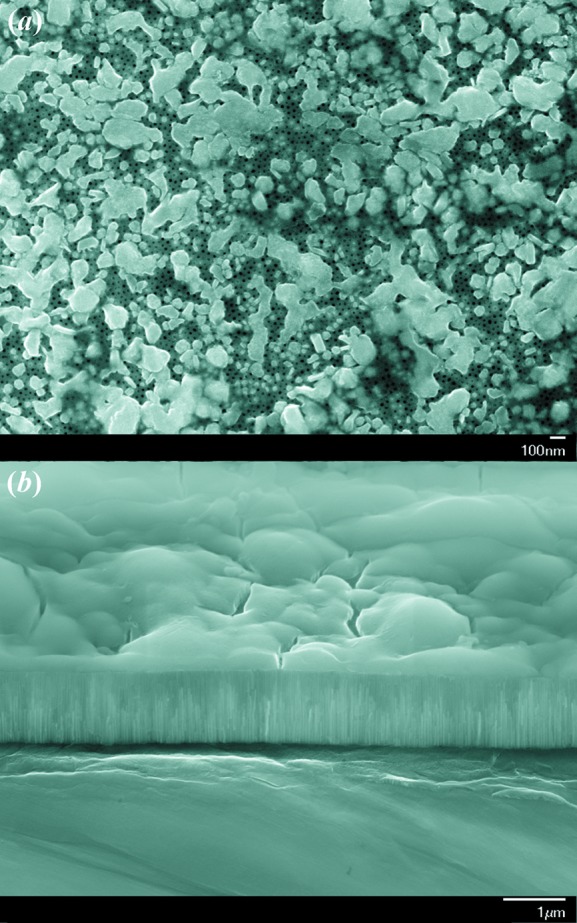
SEM images of a Co-filled porous alumina template. (*a*) The pores can be seen as dark points in the top view. They are partly overfilled with Co, so that Co islands are formed on the surface. (*b*) Cross section of the same sample as (*a*). The nanorods are visible as bright parallel pillars.

The pores were filled with Co by pulsed electrodeposition (Nielsch *et al.*, 2000 ▶) from an aqueous solution composed of 0.3 *M* CoSO_4_·7H_2_O and 45 g l^−1^ H_3_BO_3_ at room temperature and a pH value of 6.4 (Ramazani *et al.*, 2012 ▶). Such a Co-filled alumina template observed with scanning electron microscopy (SEM) is shown in Fig. 1 ▶. As can be seen in Fig. 1 ▶(*b*), the pores were not homogeneously filled up to the level of the surface.

**Figure 2:**
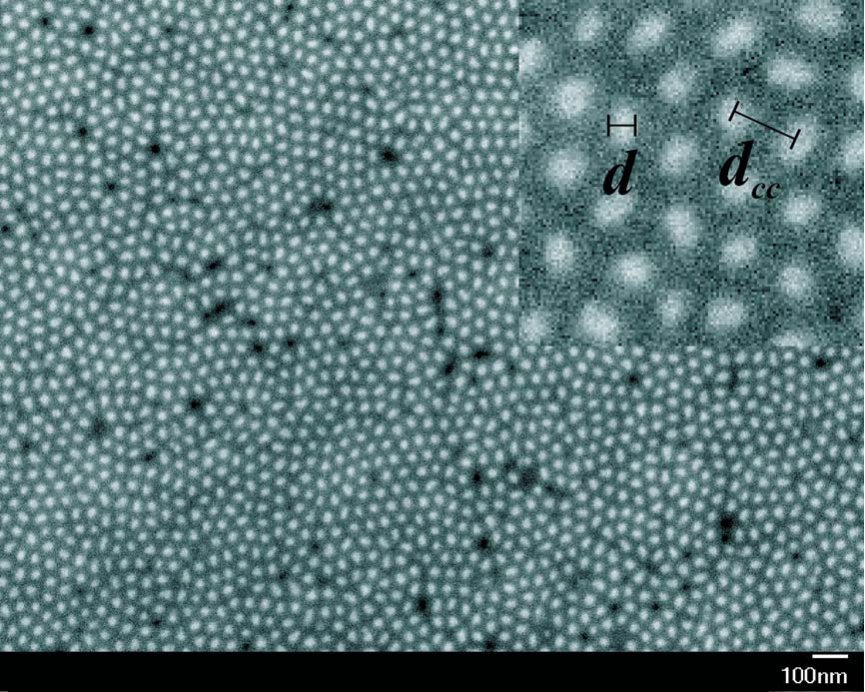
SEM top view of the etched Co nanorod array. The white circles are the end faces of the nanorods, while the dark ones represent empty pores. Upper right inset: magnified image revealing the rod diameter *d* and the centre-to-centre distance 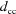.

As a consequence, it was necessary to remove alumina (and partly Co) in order that most of the nanorods end at the alumina surface. This was realized by an etching process, which was performed with an Ar-ion beam milling system (Leica EM RES101) under etching conditions of 6 kV voltage, 2.2 mA current and 30° milling angle. Owing to sample oscillation during the etching process, an area with a diameter of ∼8 mm could be homogeneously etched. In Fig. 2 ▶ the top view of the etched Co sample is shown. The white circles represent the cross-sectional areas of the nanorods, which sit flush with the alumina surface. The nanorods with average diameter 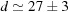
 nm and length 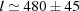
 nm are hexagonally arranged with a centre-to-centre distance of 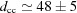
 nm (see Fig. 2 ▶).

**Figure 3:**
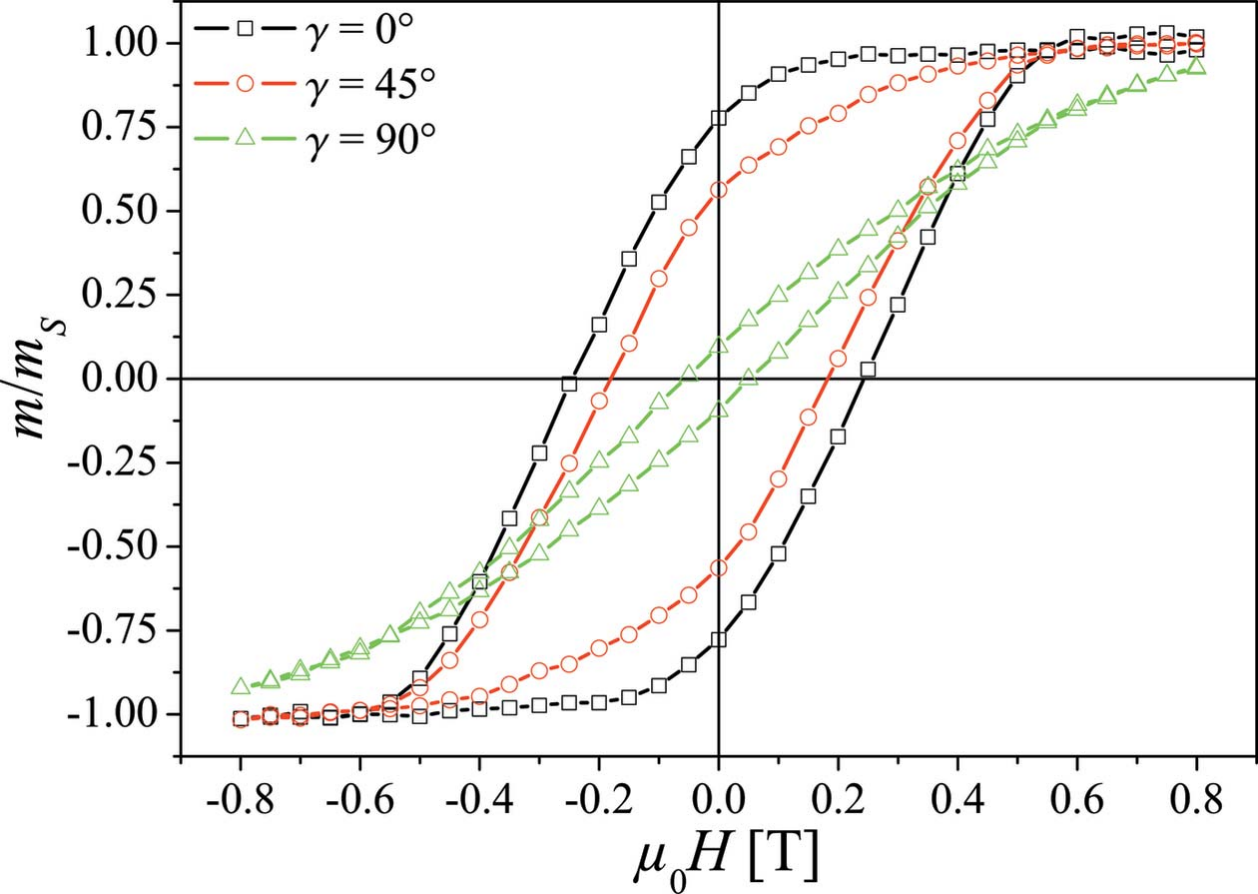
Magnetization measurements of the Co nanorod array, with γ being the angle between the applied magnetic field **H** and the long rod axes.

Magnetic characterization of the array was carried out using a vibrating sample magnetometer (VSM, LakeShore VSM 7400). The magnetization loops were recorded at room temperature for different angles γ between the magnetic field 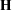 and the long rod axes in the field range from −0.8 to +0.8 T (see Fig. 3 ▶).

The magnetization measurements reveal that the Co nanorod array exhibits an effective anisotropy (due to magnetocrystalline and shape anisotropy) with the easy axis along the long rod axis (Ramazani *et al.*, 2012 ▶; Srivastav & Shekhar, 2014 ▶).

### 2.2. SANS experiment   

**Figure 4:**
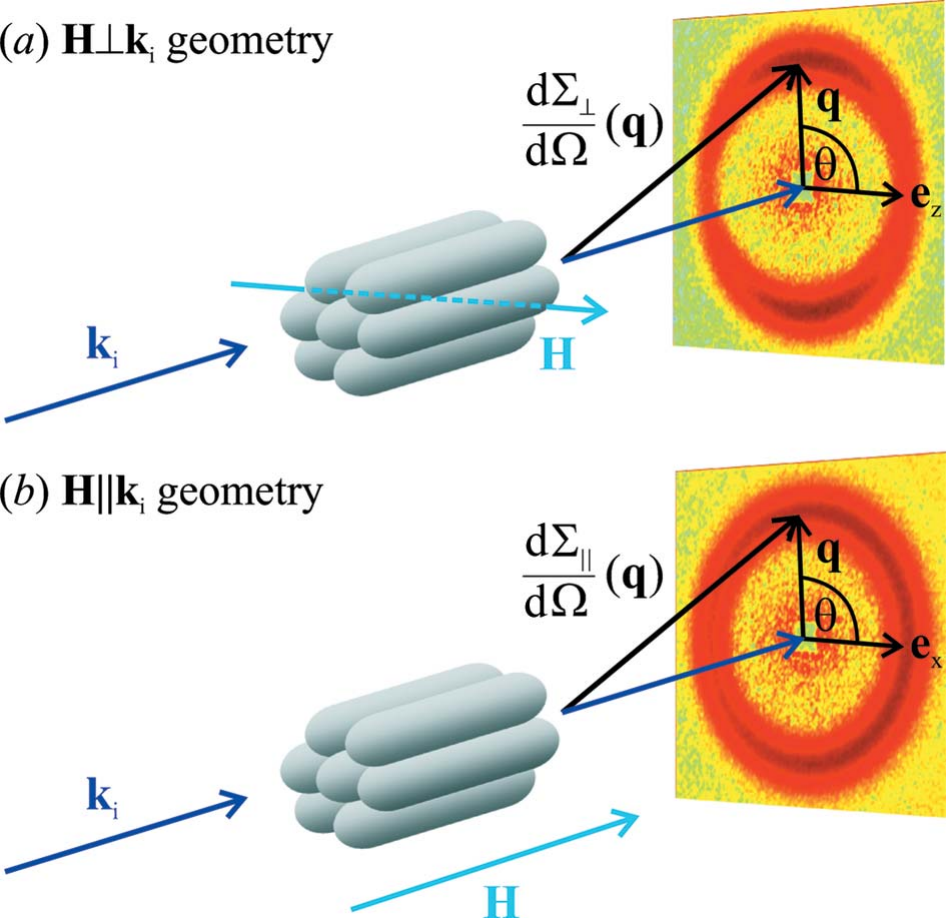
The two different scattering geometries for magnetic field dependent SANS. (*a*) **H** 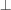 **k** 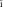 geometry: the long rod axes are aligned parallel to the incident neutron beam 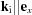 and perpendicular to the applied magnetic field **H**. (*b*) **H** 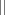 **k** 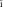 geometry: the long rod axes are aligned parallel to the incident neutron beam 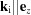 and parallel to the applied magnetic field **H**. With reference to equations (1) and (2) we emphasize that in both geometries the applied-field direction 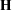 defines the 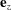 direction of a Cartesian laboratory coordinate system and that 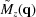 denotes the respective longitudinal magnetization Fourier coefficient, while 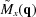 and 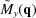 are the respective transverse components, varying in the 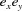 plane. The angle θ specifies the orientation of the scattering vector on the two-dimensional detector; it is measured between 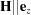 and 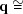 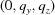 (*a*) and between 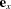 and 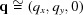 (*b*).

SANS experiments were performed at KWS-1 (Jülich Centre for Neutron Science, Outstation at MLZ, Garching, Germany), at V4 (Helmholtz-Zentrum Berlin, Germany) and at the D33 instrument at the Institut Laue–Langevin (ILL, Grenoble, France); here, we only show ILL data. At ILL, we used unpolarized incident neutrons with a mean wavelength of 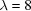
 Å [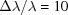
% (FWHM)] and two sample-to-detector distances of 12.8 and 2.5 m, resulting in an accessible *q* range of 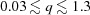
 nm^−1^. Magnetic field dependent measurements were carried out by first applying a large positive field (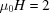
 T), which is assumed to saturate the sample (compare Fig. 3 ▶), and then reducing the field to the experimental value (following the magnetization curve). This procedure was executed for two different scattering geometries, namely **H** 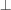 **k** 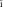 geometry (Fig. 4 ▶ *a*) and **H** 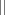 **k** 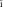 geometry (Fig. 4 ▶ *b*). All data were collected at room temperature. SANS data reduction (correction for background scattering, transmission, detector efficiency) was carried out using the *GRAS_ans_P* software package (Dewhurst, 2001 ▶).

## 3. SANS cross sections   

For the scattering geometry where the applied magnetic field **H** 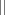 **e**_*z*_ is perpendicular to the wavevector **k** 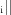 **e**_*x*_ of the incoming neutron beam (**H** 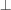 **k** 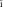), the unpolarized elastic differential SANS cross section 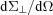 of a ferromagnet can be written as (Michels & Weissmüller, 2008 ▶)
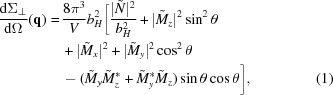
whereas for **H** 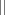 **k** 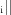 **e**_*z*_ one obtains (Michels *et al.*, 2011 ▶)
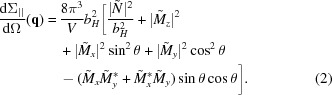
In equations (1) and (2), *V* denotes the scattering volume, 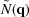 is the nuclear scattering amplitude, and 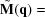 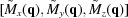 represents the Fourier coefficient of the magnetization 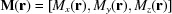; the asterisks ‘
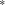
’ mark the complex-conjugated quantity. The atomic magnetic form factor 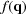 in the expression for the atomic magnetic scattering length 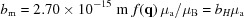 was set to unity, which is permissible along the forward direction (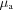: atomic magnetic moment; 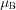: Bohr magneton). The above relation 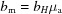 defines the quantity 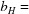 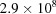
 A
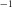
 m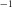, which is independent of the material (Michels & Weissmüller, 2008 ▶); 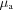 was absorbed into the expression for the saturation magnetization 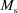, which enters the expression for the Fourier coefficients. Note that 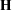 is assumed to be parallel to 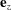 in both geometries, so that 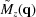 in both equations (1) and (2) denotes the corresponding longitudinal magnetization Fourier coefficient, while 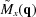 and 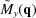 are the respective transverse components, giving rise to spin-misalignment scattering. For **H** 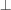 **k** 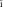, the angle θ is measured between 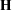 and 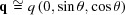, whereas for **H** 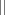 **k** 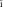, θ is the angle between 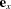 and 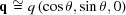 (compare Fig. 4 ▶).

At magnetic saturation, when the magnetization of the rods is perpendicular (**H** 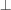 **k** 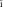) or parallel (**H** 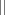 **k** 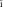) to the rod axes, equations (1) and (2) reduce to
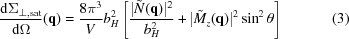
for **H** 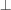 **k** 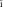 and to
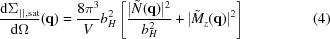
for **H** 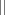 **k** 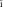.

## 4. Results and discussion   

**Figure 5:**
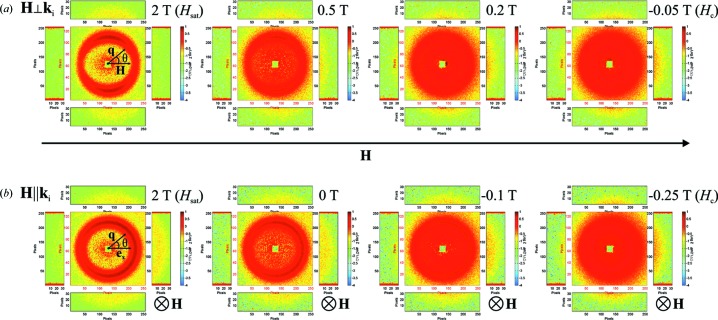
SANS cross sections 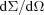 on the two-dimensional area detector for selected applied magnetic fields (see insets) (logarithmic colour scale). (*a*) **H** 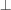 **k** 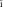; (*b*) **H** 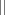 **k** 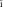.

The experimental differential SANS cross sections 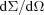 of the Co nanorod array for the two scattering geometries are shown in Fig. 5 ▶ for selected applied magnetic fields between saturation (left images) and the respective coercive fields (right images).

At saturation in **H** 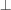 **k** 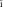 geometry, an intensity ring occurs with maxima perpendicular to **H** (seen as two dark-red half-moons; Fig. 5 ▶ *a*, left). With decreasing magnetic field, scattering due to transverse spin components emerges at smaller *q* (see below) and a maximum (overall) intensity can be observed at the coercive field 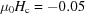
 T (Fig. 5 ▶ *a*, right). The same qualitative behaviour is detected in **H** 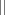 **k** 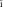 geometry (Fig. 5 ▶ *b*), except that the scattering at saturation (Fig. 5 ▶ *b*, left) is isotropically distributed on the ring.

The intensity rings that occur in both scattering geometries arise from the fact that the hexagonal order of the rods is not perfect over the whole scattering (coherence) volume, but is rather restricted to domains with a size of a few hundred nanometres (see Fig. 2 ▶). This gives rise to Debye–Scherrer diffraction rings. The half-moon intensity maxima in **H** 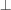 **k** 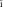 geometry reflect the angular anisotropy of the SANS cross section at saturation, which follows the well known 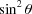 dependence [compare equation (3) and the discussion below]. By contrast, for the **H** 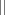 **k** 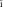 geometry, the SANS cross section at saturation exhibits an isotropically distributed intensity, *i.e.* 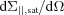 depends only on the magnitude *q* of the scattering vector 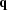; the slight intensity asymmetry that can be detected in Fig. 5 ▶(*b*) is due to a small misalignment of the sample relative to the incident beam. By comparison to equation (4), isotropy of 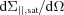 implies that the sum of 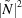 and 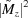 is isotropic. In the later data analysis, we will assume that both Fourier coefficients are isotropic (see below).

**Figure 6:**
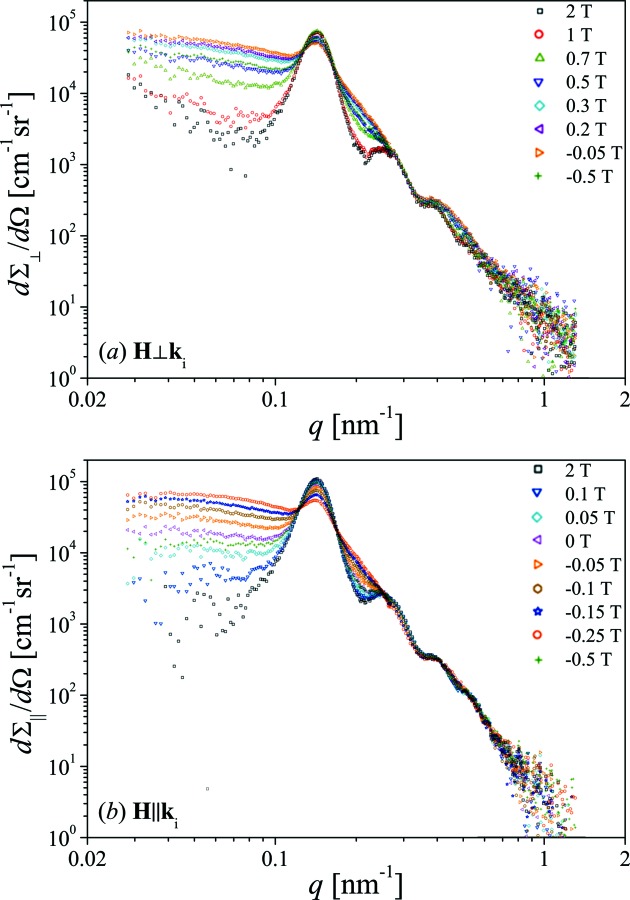
Radially averaged scattering cross sections 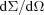 as a function of *q* and at selected applied magnetic fields *H* (see insets) for (*a*) **H** 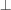 **k** 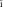 geometry and (*b*) **H** 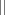 **k** 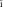 geometry (log–log scale).

The resulting radially averaged data of the differential SANS cross sections of the Co nanorod array are displayed in Fig. 6 ▶. The intensity rings observed in both geometries on the two-dimensional detector images at 2 T can be identified in the radially averaged data (black open squares in Fig. 6 ▶) as the low-*q* peak at 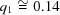
 nm^−1^ (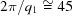
 nm). Moreover, two additional peaks were detected at higher *q* values (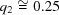
 nm^−1^ and 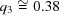
 nm^−1^), which can also be related to the hexagonal short-range order of the rods.

**Figure 7:**
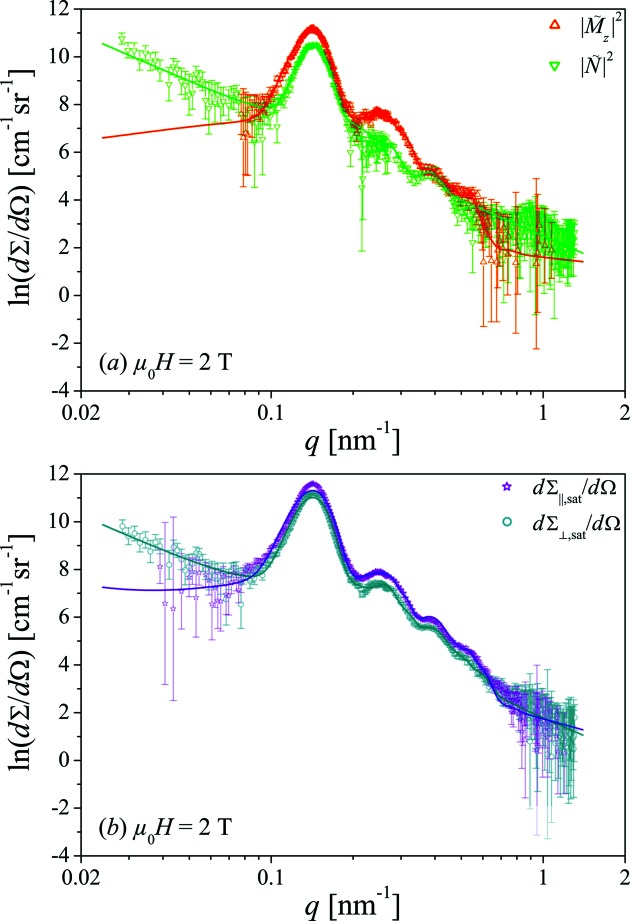
(*a*) Nuclear 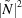 and longitudinal magnetic 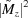 scattering cross sections as well as (*b*) 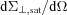 and 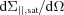 as functions of *q*; note that the logarithm of 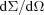 is plotted on a linear scale *versus q* on a logarithmic scale. Solid lines are data fits to equation (7).

Before discussing the field dependence of 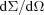, we provide an analysis of the SANS data in the saturated state. For fully saturated particles, like the Co nanorod array under study at a magnetic field of 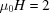
 T, equations (1) and (2) reduce to equations (3) and (4). We now assume that both Fourier coefficients 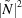 and 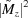 are independent of the orientation of 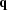 [as supported by the two-dimensional data shown in Figs. 5 ▶(*a*) and (*b*)]. Radial averaging of the scattering cross section at saturation in **H** 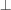 **k** 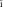 geometry [equation (3)] then results in 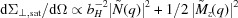, whereas for **H** 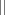 **k** 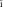 geometry we obtain 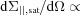 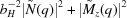. By assuming that 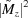 at saturation is independent of the orientation of the externally applied magnetic field, one can combine these two equations and separate the nuclear from the longitudinal magnetic SANS:
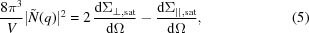


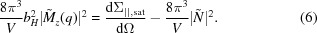
The so-determined experimental nuclear 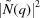 and longitudinal magnetic 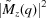 SANS cross sections are shown in Fig. 7 ▶(*a*); for simplicity, we will omit the constant prefactors 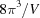 and 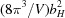 in the following.

For the quantitative description of 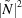 and 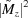 as well as the SANS data at saturation (Fig. 7 ▶ *b*), we consider a magnetic field independent model,
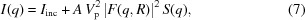
where 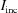 denotes the incoherent scattering background, *A* is a scaling constant, which is proportional to the particle density and the respective scattering-length density contrast, 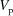 is the particle volume, and 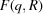 is the form factor of a cylinder for **q** being perpendicular to the long rod axes; 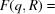 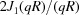, where 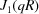 is the spherical Bessel function of first order with 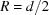 being the rod radius. The structure factor is modelled as a sum of Gaussians, 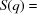 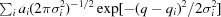, with the Bragg peak positions given by the two-dimensional hexagonal lattice at 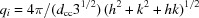, where (*hk*) = (10), (11), (20), (21), (30) and (22).

The data fits by this model with 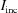, *A*, 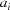, 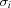, 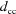 and *R* as adjustable parameters are shown as the solid lines in Fig. 7 ▶. Obviously, the considered model, equation (7), does provide an excellent description of the measurements. The resulting values of the structural fit parameters are listed in Table 1 ▶ and are in good agreement with each other as well as being consistent with the results from electron microscopy, where we have found 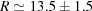
 nm and 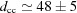
 nm.

The magnetic scattering contribution 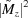 is larger than the nuclear SANS 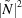 (see Fig. 7 ▶ *a*), and the averaged experimental ratio 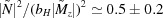 is in good agreement with the theoretically calculated value of the nuclear-to-magnetic scattering-length density contrasts 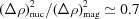. For the computation of the latter, we used 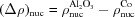 with 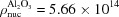
 m^−2^ and 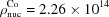
 m^−2^, and 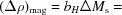 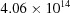
 m^−2^ with 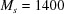
 kA m^−1^ for Co (Skomski, 2003 ▶) and 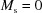 for the nonmagnetic Al_2_O_3_ matrix. This finding suggests that the nuclear and magnetic form factors of the nanorods are not too different from each other, in agreement with the observations in Fig. 7 ▶(*a*) and the fit results listed in Table 1 ▶.

Let us now discuss the field dependence of 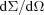. By reducing the field from the saturation value of 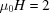
 T to smaller fields, the total nuclear and magnetic SANS cross sections 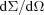 in both scattering geometries increase at smaller 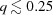
 nm^−1^, and the total intensity in the first Bragg peak is slightly reduced and washed out (compare Fig. 6 ▶). The intensity increase continues until the coercive fields (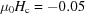
 T in **H** 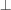 **k** 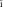 geometry and 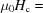 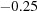
 T in **H** 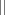 **k** 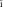 geometry) are reached. Further reduction of the fields to more negative values leads again to a decrease of the scattering intensity (see data at 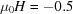
 T in Fig. 6 ▶).

The conventional ‘standard’ expression for describing magnetic SANS data of magnetic nanoparticles that are embedded in a homogeneous nonmagnetic matrix considers the particles to be homogeneously (or stepwise homogeneously) magnetized (Heinemann *et al.*, 2000 ▶; Wagner & Kohlbrecher, 2005 ▶; Wiedenmann, 2005 ▶; Disch *et al.*, 2012 ▶). The possible continuous spatial dependence of the magnetization 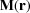 of the particles is ignored. For a dilute assembly of *N* monodisperse magnetic nanoparticles in the scattering volume *V*, the magnetic part of the total unpolarized SANS cross section is usually expressed as (Heinemann *et al.*, 2000 ▶; Wagner & Kohlbrecher, 2005 ▶; Wiedenmann, 2005 ▶; Disch *et al.*, 2012 ▶)
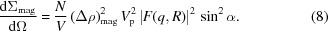
The only dependency on the applied magnetic field in equation (8) is contained in the function 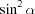, which takes into account the dipolar character of the neutron–magnetic interaction (Halpern & Johnson, 1939 ▶; Shull *et al.*, 1951 ▶). One may also include a structure factor in equation (8) [compare equation (7)], but (for rigid nanoparticles in a rigid matrix) this would only affect the *q* dependence of the scattering (similar to a particle-size distribution), not its field dependence. We also note that different definitions regarding the angle α can be found in the literature (Shull *et al.*, 1951 ▶; Heinemann *et al.*, 2000 ▶; Wagner & Kohlbrecher, 2005 ▶; Wiedenmann, 2005 ▶; Disch *et al.*, 2012 ▶).

If α is taken to be the angle between 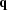 and the local direction of the magnetization 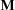 of a uniformly magnetized nanoparticle, then, for **H** 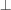 **k** 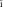 geometry, the expectation value of the function 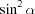 varies between a value of 1/2 at saturation and a value of 2/3 in the demagnetized state; for **H** 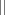 **k** 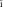, the expectation value of 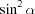 varies between a value of 1 at saturation and a value of 2/3 in the demagnetized state (Halpern & Johnson, 1939 ▶; Shull *et al.*, 1951 ▶). In other words, the above definition of α in combination with the standard expression for the SANS cross section of (dilute) nanoparticles, equation (8), can only explain an intensity increase by a factor of 4/3 (between saturation and the case of random domain orientation) in **H** 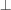 **k** 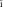 geometry, whereas it predicts an intensity decrease with decreasing field for **H** 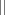 **k** 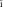. This is, however, inconsistent with the experimental observations in this work.

The measured radially averaged SANS cross sections in **H** 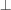 **k** 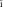 geometry change at least by a factor of 4 at 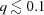
 nm^−1^ with decreasing applied magnetic field (see Fig. 6 ▶ *a*); in the ‘pocket’ at 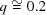
 nm^−1^ the scattering changes by a factor of about 5. For **H** 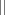 **k** 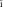 geometry, the situation is even more striking, since here we observe an intensity increase (at least by a factor of 8 at small *q*) with decreasing field (see Fig. 6 ▶ *b*).

As mentioned before, the obvious reason why equation (8) is not suited for describing the magnetic field dependent SANS cross section of the Co nanorod array is related to the fact that it describes magnetic scattering from homogeneously magnetized domains (particles). For magnetic microstructures where the magnetization vector field depends on the position 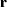 inside the sample, *i.e.* 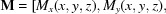 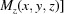, the corresponding SANS cross sections are given by equations (1) and (2), where the angle θ specifies the orientation of the scattering vector on the two-dimensional detector. Besides its spatial dependence, 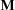 depends of course on the applied magnetic field, the magnetic interaction parameters and the details of the microstructure.

At saturation, equations (1) and (2) reproduce the 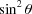 anisotropy (**H** 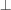 **k** 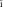) and the isotropic scattering pattern (**H** 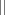 **k** 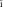) (Fig. 5 ▶). At lower fields, spin-misalignment SANS with related transverse Fourier coefficients 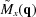 and 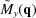 contributes to the total 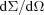, and, at least for bulk ferromagnets, may give rise to a variety of angular anisotropies (Michels *et al.*, 2006 ▶, 2014 ▶; Döbrich *et al.*, 2012 ▶). In Fig. 5 ▶, the spin-misalignment SANS is observed as the intensity that emerges with decreasing field at the smallest *q* values. The analysis of the SANS data at saturation suggests an average nanorod diameter of about 30 nm. The existence of intraparticle spin misalignment would then give rise to magnetic SANS at 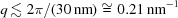, in agreement with our observations in Fig. 6 ▶. We note that in nanocrystalline bulk ferromagnets the field dependence of spin-misalignment SANS can be several orders of magnitude between a field close to saturation and the coercive field (Honecker *et al.*, 2011 ▶; Bick, Honecker *et al.*, 2013 ▶; Bick, Suzuki *et al.*, 2013 ▶; Honecker *et al.*, 2013 ▶).

The origin of the spin misalignment within the individual Co nanorods, which gives rise to the strong field dependence of 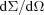, may be related to the polycrystalline nature of the rods: besides the dipolar shape anisotropy, which prefers an alignment of 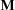 along the long rod axis, there are magnetocrystalline and magnetoelastic anisotropies (due to stress-activate microstructural defects) which give rise to internal spin disorder. Additionally, the magnetostatic stray field that emerges from neighbouring rods may produce inhomogeneous spin structures inside a given rod. A rigorous calculation of the magnetization distribution of such a nanorod array (and of the ensuing magnetic SANS) by means of numerical micromagnetics (Hertel, 2001 ▶; Nielsch *et al.*, 2002 ▶; Zighem *et al.*, 2011 ▶; Kulkarni *et al.*, 2013 ▶; Bran *et al.*, 2013 ▶) is a very complicated problem and is beyond the scope of this paper.

## 5. Summary and conclusion   

We have reported the results of magnetic field dependent unpolarized SANS experiments on a Co nanorod array. Measurement of the SANS cross section 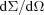 in a saturating applied field of 2 T for two different scattering geometries (**H** 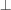 **k** 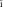 and **H** 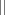 **k** 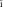) allows us to separate nuclear from magnetic SANS without employing the usual sector averaging in unpolarized SANS. The ratio of the experimentally determined nuclear-to-magnetic scattering is in good agreement with the theoretically expected value. The total SANS data in the saturated state (as well as the corresponding nuclear and magnetic contributions) could be well described by a model that combines a structure factor with the form factor of a cylinder. The obtained structural parameters (cylinder radius and centre-to-centre distance) of the Co nanorod array are consistent with the results from electron microscopy. Between 2 T and the respective coercive fields, we observe a relatively strong field dependence of 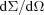, for instance, by a factor of 4 for **H** 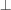 **k** 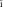. This cannot be explained by the standard expression for 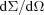, which assumes uniformly magnetized domains. It seems obvious that the strong field dependence of 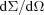 is related to intraparticle spin misalignment.

## Figures and Tables

**Table 1:** Resulting structural parameters obtained by fitting equation (7) to the nuclear 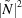 and longitudinal magnetic 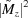 SANS cross sections as well as to the SANS data at saturation 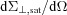 and 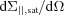 *R* denotes the rod radius and 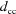 the centre-to-centre distance of the rods in the alumina layer.

	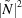	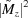	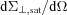	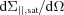
*R* (nm)	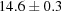	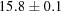	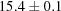	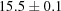
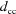 (nm)	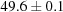	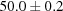	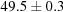	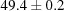
